# Imaging of the skull base and orbital tumors

**DOI:** 10.1007/s11604-024-01662-9

**Published:** 2024-09-20

**Authors:** Masafumi Sakai, Takashi Hiyama, Hirofumi Kuno, Tatsushi Kobayashi, Takahito Nakajima

**Affiliations:** 1 Department of Radiology, Institute of Medicine, University of Tsukuba, 1-1-1 Tennodai, Tsukuba, Ibaraki 305-8575 Japan; 2 Department of Diagnostic Radiology, National Cancer Center Hospital East, 6-5-1 Kashiwanoha, Kashiwa, Chiba 277-8577 Japan

**Keywords:** Orbital tumors, Skull base tumors, Orbit, Skull base, Magnetic resonance imaging (MRI), Computed tomography (CT)

## Abstract

The skull base and orbit have complicated anatomical structures where various tumors can occur. The tumor may present with neurological symptoms; however, its diagnosis is clinically difficult owing to accessibility issues. Therefore, diagnostic imaging is crucial in assessing tumors in the skull base and orbit and guiding subsequent management. Notably, some tumors have a predilection for a specific site of origin, and identifying the site of origin on imaging can help narrow the differential diagnosis. At the skull base, chordomas typically occur in the clivus, chondrosarcomas in the paramedian areas, paragangliomas in the jugular foramen, neurogenic tumors, and perineural spread in the neural foramen. Among orbital tumors, cavernous hemangiomas usually occur in the intraconal space, and pleomorphic adenomas and adenoid cystic carcinomas occur in the lacrimal glands. Some skull base and orbital tumors exhibit distinctive imaging features. Chordomas and chondrosarcomas of the skull base show high signal intensities on T2-weighted images, with chondrosarcomas often displaying cartilaginous calcifications. Paragangliomas are characterized by their hypervascular nature. In the orbit, cavernous hemangiomas and pleomorphic adenomas present unique dynamic patterns. Immunoglobulin G4-related disease forms lesions along the nerves. Identifying the tumor origin and its imaging characteristics can help narrow the differential diagnosis of skull base and orbital tumors.

## Introduction

The skull base and orbit are adjacent, complex anatomical structures. The orbit is located inferior to the anterior skull base and continuous with the middle skull base posterior to the orbit. They are also borderline and overlapping areas in neuroradiology and head and neck radiology. Notably, various tumors can occur in both areas. Tumors may present with neurological symptoms; however, the diagnosis is clinically challenging because of the difficulty in obtaining a biopsy. Diagnostic imaging is crucial in narrowing the diagnosis of tumors in the skull base and orbit and determining subsequent management. It can narrow the diagnosis of tumors based on the site of occurrence and the characteristic imaging findings. Therefore, in this article, we review the common skull base and orbit diseases and explain the clinical and imaging findings and differential diseases.

### Skull base

The skull base is the base of the neurocranium, which contains the brain. It contains numerous foramina, which are pathways for the nerves and vessels that pass inside and outside the neurocranium. The skull base is bordered by ridges and divided into anterior, middle, and posterior ones.

Skull base tumors are divided into three categories: lesions that originate in the skull base, those that extend from the intracranial space, and those that extend from the head and neck, such as the paranasal sinuses, nasopharynx, and orbit. Notably, some tumors have a specific predilection site (Table 1). These include chordomas in the median of the middle and posterior skull bases, chondrosarcomas in the paramedian of the middle and posterior skull bases, and paragangliomas in the jugular foramen. Neurogenic tumors and perineural spread are observed in the neural foramen. Therefore, presuming the tumor location from the imaging findings narrows the differential diagnosis.

**Table 1:** Skull base tumors with predilection site of origin

Skull base tumor	Predilection site	Imaging findings
Chordoma	Clivus (sphenooccipital synchondrosis)	High T2 signal Lower apparent diffusion coefficient (ADC) values than those of chondrosarcomas
Chondrosarcoma	Temporooccipital junction, clivus	Ring or arc shapes calcification High T2 signal Higher ADC values than those of chordomas Whorls or a honeycomb pattern of enhancement
Neurogenic tumor, perineural spread	Neural foramen (foramen ovale, foramen rotundum, jugular foramen, and so on.)	Enlargement of the neural foramen Localization of lesions along the nerve
Paraganglioma	Jugular foramen	Enlargement and erosion of the jugular foramen Salt and pepper appearance Early enhancement Markedly increased blood flow
Bone metastasis	Clivus, apex of petrosal bone, sphenoid bone	Osteolytic and osteogenic changes Low T1 signal in the bone marrow Restricted diffusion

### Chordoma

Chordoma arises from the residual notochord and, therefore, occurs along the body’s centerline. It typically occurs in the skull base (38.7%), sacrococcygeal region (34.3%), and spine (27.0%) [1]. Diagnosis is usually made in individuals aged ≥ 60 years; however, it also occurs in younger individuals, including pediatric patients [2]. The tumor’s growth is slow, and symptoms appear gradually. The most common initial symptoms are headache and diplopia due to cranial nerve involvement [3], and surgical resection is the standard treatment for this condition. However, complete resection is difficult, and postoperative radiation therapy is recommended [2].

**Fig. 1:**
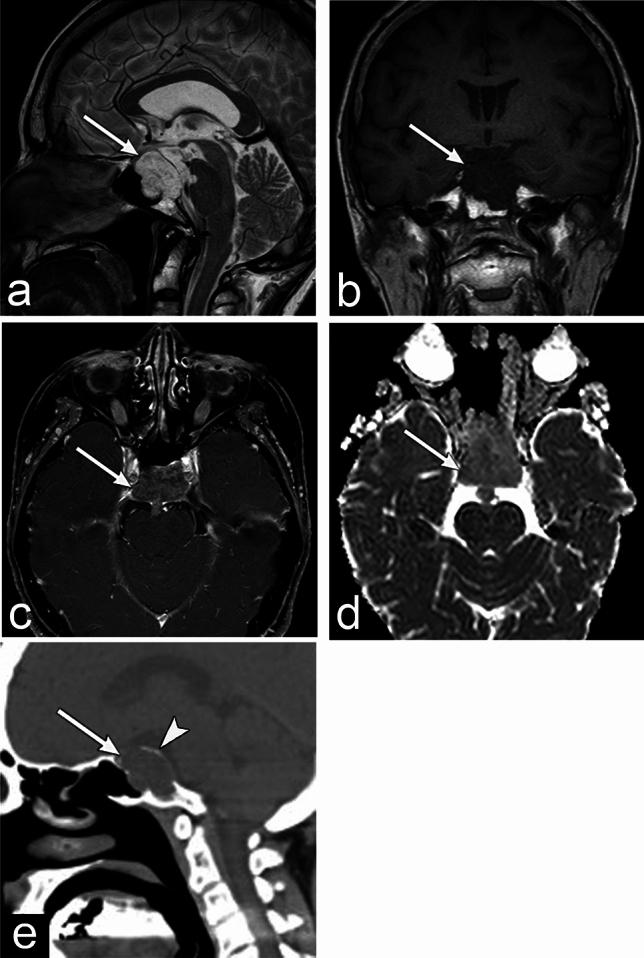
Chordoma of a woman in her 50s with bilateral diplopia. **a**–**d** MRI shows a well-defined lobulated mass (arrow) protruding upward from the midline of the clivus. The mass shows slightly heterogeneous high signal intensity on the T2 weighted image (WI) and homogeneous low signal intensity on T1WI with heterogeneous mild enhancement. The apparent diffusion coefficient value is 1.11 × 10^–3^ mm^2^/s. **e** CT shows a low attenuation mass (arrow) with destruction of the clivus. Lenear high attenuation structure (arrowhead), which appears to be residual bone, is observed at the margins of the mass

Chordoma occurs predominantly in the median part of the clivus and the sphenooccipital synchondroses. CT scans shows a well-defined, expansile mass with bone destruction (Fig. 1) [3]. Calcification reflecting residual destructive bone may be observed on CT [3]. MRI typically shows low-to-intermediate signal intensity on the T1 weighted image (WI); however, areas of high signal intensity may reflect hemorrhage and mucin (Fig. 1) [3]. T2WI shows high signal intensity due to the myxoid matrix and physaliphorous cells that contain a large amount of fluid. However, low signal areas reflecting calcification, hemorrhage, protein-rich fluid, and septa are also observed [3]. Contrast enhancement is often moderate to marked; however, few contrast enhancements are occasionally observed, reflecting necrosis or mucin in the tumor [3]. The differential diagnoses include nasopharyngeal carcinoma, sphenoid sinus carcinoma, pituitary neuroendocrine tumor, metastatic bone tumor, fibrous dysplasia, and chondrosarcoma. Notably, chondrosarcomas have similar imaging findings and often require a differential diagnosis. The first difference is that chondrosarcomas show higher apparent diffusion coefficient (ADC) values (2.07–2.44 × 10^–3^ mm^2^/s) than chordomas (1.30–1.33 × 10^–3^ mm^2^/s) [4, 5]. The second difference is the occurrence site. Chondrosarcomas often originate from the tempero-occipital junction and paramedian area of the skull base, whereas chordomas occur in the median area of the skull base. Chordomas show high signal intensity on T2WI and are centered in the clivus, differentiating it from nasopharyngeal and sphenoid sinus carcinoma. Since chordomas have a rare prevalence of lymph node metastasis, retro-pharyngeal adenopathy suggests nasopharyngeal carcinoma [6].

### Chondrosarcoma

Chondrosarcoma is a malignant tumor that arises from the endochondral cartilage remnants of the synchondroses. At the skull base, it frequently occurs in the temporo-occipital junction (66%), clivus (28%), and sphenoethmoid complex (6%) [7]. It can be associated with Paget disease, Ollier disease, and Maffucci syndrome. Ollier disease and Maffucci syndrome are enchondromatoses in which multiple enchondromas occur in bones throughout the body [8]. Affected patients are mostly in their 40s and 70s, with no sex difference. Symptoms include headaches and neurological deficits, such as visual loss, diplopia, abducens nerve paralysis, and facial dysesthesia caused by compression of the cranial nerves and brain stem [8]. Treatment includes resection and adjuvant radiotherapy.

**Fig. 2:**
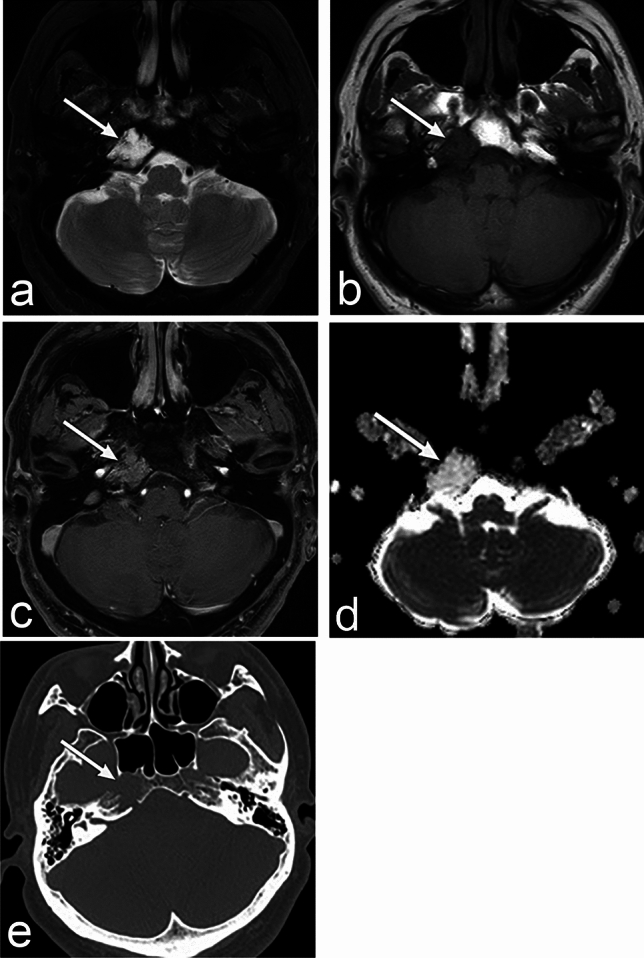
Chondrosarcoma of a man in his 50s with right diplopia. **a**–**d** MRI shows a well-defined mass (arrow) in the right temporo-occipital junction. The mass shows homogeneous high signal intensity on T2 weighted image (WI) and homogeneous low signal intensity on T1WI with heterogeneous moderate enhancement. The apparent diffusion coefficient value is as high as 1.89 × 10^–3^ mm^2^/s. **e** CT shows a mass (arrow) with the destruction of the clivus without calcification

On CT, chondrosarcoma is an isodense to hyperdense mass with heterogeneously enhanced bone erosion (Fig. 2), and calcification with ring or arc shapes is observed in 50% of cases [9]. MRI shows high signal intensity on T2WI and low signal intensity on T1WI, with "whorls" or a "honeycomb" pattern of enhancement [9]. Differential diagnoses include chordoma, bone metastases, myeloma, nasopharyngeal carcinoma, cholesterol granuloma of the petrous apex, and chondromyxoid fibroma.

### Trigeminal schwannoma

Schwannomas are benign tumors derived from the Schwann cells of peripheral nerves. In the skull base, it can occur in the cranial nerves (CN) III-XII [8]. CN VIII is the most common, followed by CN V. Trigeminal schwannomas in the skull base occur mostly in the cisterna to Meckel’s cave, the foramen rotundum, and the foramen ovale. Trigeminal schwannomas account for 1–2% of intracranial schwannomas and are predominantly observed in patients in their 40s–60s [10]. The most typical symptoms are trigeminal neuropathy and facial pain [10].

**Fig. 3:**
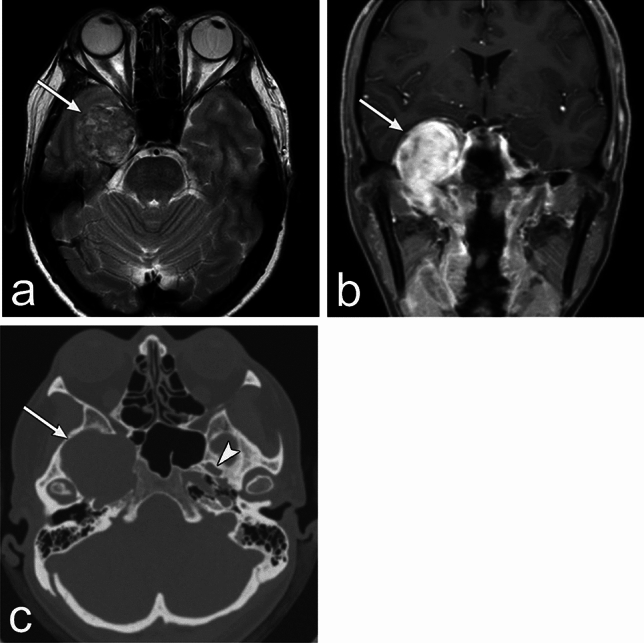
Trigeminal schwannoma of an asymptomatic woman in her 50s. The lesion is identified incidentally. **a**, **b** MRI shows a well-defined mass (arrow) in the right Meckel’s cave and right foramen ovale with a fusiform appearance along the nerve. The mass shows heterogeneous signal intensity with an equal to high signal compared to the brain parenchyma on T2 weighted image (WI). The mass shows heterogeneous enhancement. **c** CT shows dilatation of the right foramen ovale (arrow). The arrowhead indicates a normal left foramen ovale

Schwannoma is a well-defined mass with a fusiform appearance along the deriving nerve (Fig. 3). Histologically, the Antoni type A region shows an intermediate signal intensity on T2WI, whereas the Antoni type B region shows a high signal intensity on T2WI. Schwannomas show homogenous enhancement; however, they become heterogeneous as they degenerate. They may present with cystic degeneration, hemorrhage, and calcification [8]. They grow slowly and expansively within the neuroforamen; therefore, they cause enlargement of the neuroforamen and bone remodeling.

### Paraganglioma

Paraganglioma is a benign tumor arising from the paraganglia. At the skull base, Glomus tympanicum tumors occur within the tympanic cavity, whereas Glomus jugulare tumors occur in the jugular foramen. The Arnold nerve, an auricular branch of the vagus nerve, is associated with the development of Glomus jugulare tumors. Most paragangliomas of the head and neck are catecholamine-nonsecretory. Approximately 35–40% of head and neck paragangliomas are associated with familial disorders, typically involving mutations in the succinate dehydrogenase gene family [11]. Glomus jugulare tumor presents with pulsatile tinnitus, hearing loss, hoarseness, and difficulty swallowing due to paralysis of CN IX-XI [12]. Previously, the treatment included mainly surgery and radiotherapy. Recently, however, active surveillance is increasingly selected to avoid the complications of surgery and radiation therapy, as many paragangliomas are indolent [11].

**Fig. 4:**
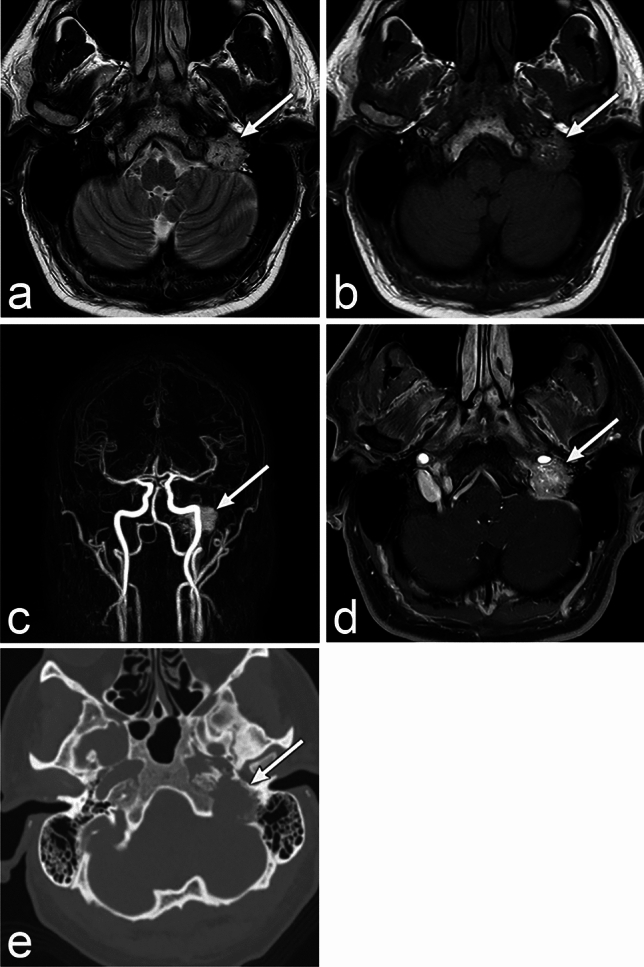
Paraganglioma of a man in his 50s with hoarseness. **a**–**d** MRI shows a well-defined mass (arrow) in the left jugular foramen. The mass shows a "salt and pepper appearance," which is a coexistence of dotted high and low signals on the T2 weighted image (WI) and T1WI. The mass shows early, marked enhancement. **e** CT shows enlargement of the left jugular foramen with bone erosion (arrow)

On MRI, paraganglioma shows low signal intensity on T1WI and high signal intensity on T2WI (Fig. 4). Furthermore, it may show a so-called "salt and pepper appearance" that is a coexistence of dotted high and low signals on T1WI and T2WI [13]. This appearance reflects the fast and slow blood flow within the tumor. Dynamic contrast studies show homogeneous early enhancement [14]. MRI perfusion shows markedly increased blood flow, which helps in differential diagnosis [15]. The Glomus jugulare tumor shows enlargement and erosion of the jugular foramen on CT [16]. It can extend into the tympanic cavity or posterior cranial fossa [14]. Gallium 68 tetraazacyclododecane tetraacetic acid–octreotate positron emission tomography (PET)/CT shows a high accumulation frequency in paragangliomas [15].

### Perineural spread

Perineural spread is a pathological condition where the tumor extends along the peripheral nerves. In the skull base, the perineural spread of head and neck cancer shows a mass formation in the neural foramen on CT and MRI. Various histologic types of head and neck tumors develop perineural spread; adenoid cystic carcinoma and squamous cell carcinoma are most common [17]. In head and neck cancers, perineural spread is most common in the trigeminal and facial nerves. The symptoms depend on the involved nerve. Symptoms include pain, paresthesia, burning sensation, diplopia, blurred vision, and weakness [17]. The onset of symptoms is often delayed, and 40% of patients are asymptomatic [17]. Therefore, imaging is crucial in diagnosing perineural spread.

**Fig. 5:**
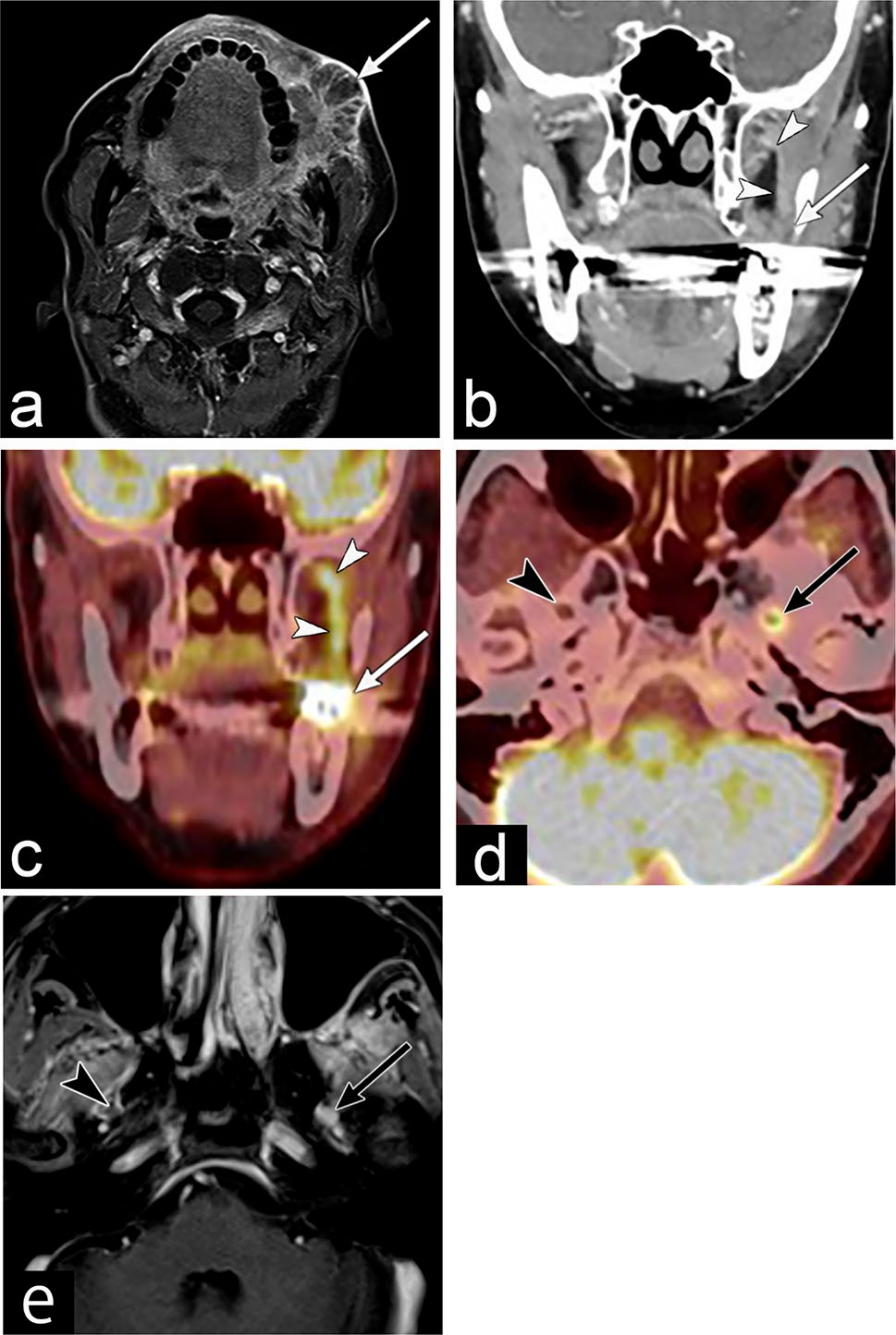
Perineural spread of buccal mucosa squamous cell carcinoma of a woman in her 50s with left buccal swelling. **a** Axial contrast-enhanced (CE) T1 weighted images (WI) with fat suppression show enhanced mass (white arrow) in the left buccal space. **b**–**d** 18F-fluorodeoxyglucose (FDG) positron emission tomography CE CT shows linear enhancement and increased FDG uptake (white arrowheads) extending upward from the left buccal mucosa mass (white arrow). This is continuous within the left foramen ovale, and shows increased FDG uptake (black arrow) within the left foramen ovale. The black arrowhead indicates a normal right foramen ovale. **e** Axial CE T1WI with fat suppression shows enhanced mass within the left foramen ovale (black arrow). The black arrowhead indicates a normal right foramen ovale

The imaging findings include enlargement and destruction of the neural foramen, loss of normal fatty tissue around the nerve, nerve enlargement and enhancement, muscle atrophy due to denervation, and projection of the lateral wall of the cavernous sinus (Fig. 5) [18]. Furthermore, 18F-fluorodeoxyglucose PET/CT shows increased linear uptake along the involved nerves and increased uptake in muscles atrophied by denervation [19]. Muscle atrophy indicates denervation, which can affect muscles such as those of mastication (mandibular nerve) and the tongue (hypoglossal nerve).

### Bone metastasis

Bone metastases to the skull base are most common in the clivus, apex of the petrosal bone, and sphenoid bone due to the abundance of bone marrow [8]. The most common primary tumors are prostate, breast, and lung cancers, as well as lymphoma [20]. Hematogenous spread is the most common route of metastasis [8]. Symptoms include pain, swelling, and neurological deficits, such as facial paralysis and hypesthesia in the trigeminal region; however, they can also be asymptomatic [8, 21].

**Fig. 6:**
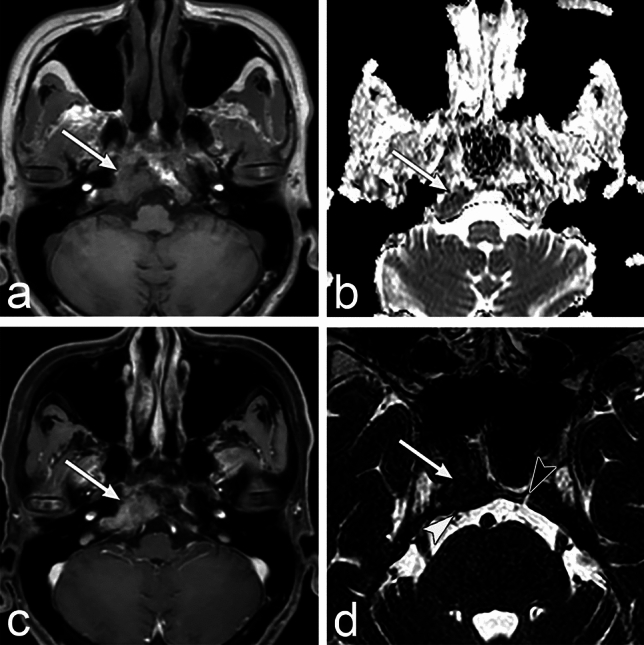
Bone metastasis from the gastric cancer of a man in his 50s with diplopia. **a** Axial T1 weighted images (WI) show mass (arrow) presenting low signal intensity on the right side of the clivus. **b** The apparent diffusion coefficient value is low in the mass (arrow) **c** Axial contrast-enhanced T1WI with fat suppression shows enhancement in the mass (arrow). **d** MR cisternography shows that the mass involved the right Dorello canal and caused diplopia. The white arrowhead indicates the right abducens nerve. The black arrowhead indicates the left Dorello canal

CT shows osteolytic and osteogenic changes. However, intertrabecular metastases cannot be detected using CT. MRI shows low signal intensity on T1WI due to the tumor’s replacement of the fatty marrow with restricted diffusion and enhancement (Fig. 6).

### Fibrous dysplasia

Fibrous dysplasia is a developmental fibro-osseous disorder in which immature bone and fibrous tissue replace the bone marrow. G-nucleotide binding protein alpha subunit activating mutations are detected in 50–70% of fibrous dysplasia cases [22]. Histopathologically, fibroblast proliferation and woven bone are observed. Malignant transformation rarely occurs, and osteosarcoma is the most common histological type [23]. It can affect any bone, including the skull base. Fibrous dysplasia is classified based on its clinical presentation as monostotic or polyostotic [24]. Syndromes associated with fibrous dysplasia include the McCune-Albright and Mazabraud syndromes. McCune-Albright syndrome presents with fibrous dysplasia, café au lait spots, and precocious puberty [25]. Mazabraud syndrome is associated with fibrous dysplasia and intramuscular myxoma. The symptoms of fibrous dysplasia depend on the lesion site. At the skull base, patients present with swelling, deformity, pain, and nerve compression causing visual disturbances, hearing loss, trigeminal neuralgia, and facial nerve palsy. Reduction surgery is performed on patients with neurological symptoms and facial deformities.

**Fig. 7:**
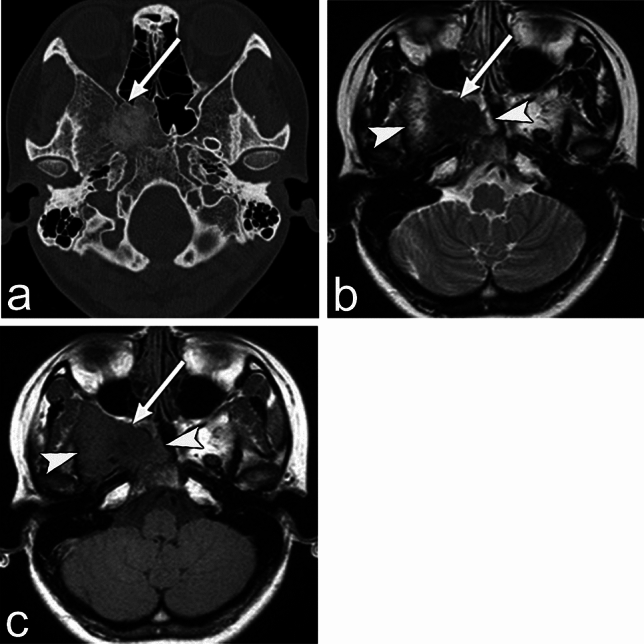
Fibrous dysplasia of an asymptomatic woman in her 20s. The lesion is identified incidentally. **a** CT shows a ground-glass appearance (arrow) with swelling of the right side of the sphenoid bone. **b**, **c** The area (arrow) of the lesion that shows a ground-glass appearance on CT shows low signal intensity on the T2 weighted image (WI) and T1WI. Other areas (arrowheads) of the lesion show high signal intensity on T2WI and mildly high signal intensity on T1WI

Radiograph and CT show a ground-glass appearance with bony expansion, and MRI shows low signal intensity on T1WI and T2WI, reflecting fibrous tissue and bony trabecular composition (Fig. 7) [8]. Cystic changes may be mixed on a radiograph and CT. MRI findings may vary due to the presence of bony trabeculae, collagen, cyst degeneration, and hemorrhage [26]. Therefore, diagnosing fibrous dysplasia may be difficult using MRI. However, low signal intensity on T2WI, which reflects fibrous tissue, is useful for diagnosis. Ossifying fibroma is a differential diagnosis that can be distinguished from fibrous dysplasia by its well-defined margin [27]. In the case of juvenile ossifying fibroma, a variant of ossifying fibroma, it has a more rapid and aggressive progression and higher recurrence rate than fibrous dysplasia [27].

### Orbit

The orbit is a skeletal cavity that houses the eyeball and its appendage, protecting them from the outside. In addition to the eyeball, the orbit contains external ocular muscles and nerves, such as the optic nerve, as well as orbital fatty tissue, lacrimal gland, lacrimal sac, blood vessels, and eyelids. Due to its adjacency to the skull base, tumors arising in one area can extend to the other. Therefore, understanding the tumors that can occur in each area is useful for differential diagnosis.

The predilection site within the orbit differs based on the tumor (Table 2). Therefore, identifying the origin of the tumor narrows the differential diagnosis. The orbit is anatomically divided into three major parts (the eyeball, intraconal space, and extraconal space) [28]. The space circumscribed by the four rectus muscles of the external ocular muscles and the eyeball is called the intraconal space. The intraconal space contains the external ocular muscles, fat, and cranial nerves, such as the optic nerve and the ciliary ganglion, the ophthalmic artery, and the vein [28]. The extraconal space contains bone, fat, and cranial nerves, such as the lacrimal and frontal branches of the ophthalmic nerve and the lacrimal gland [28]. Cavernous hemangiomas, optic gliomas, and meningiomas can occur within the intraconal space. Lacrimal gland tumors, schwannomas, and lacrimal sac cancers can occur in the extraconal space. Malignant melanomas and retinoblastomas can occur in the eyeball.

**Table 2:** Orbital tumors with predilection site of origin

Orbital tumor	Predilection site	Imaging findings
Immunoglobulin G4-related disease	Lacrimal gland, external ocular muscles, trigeminal nerve	Relatively low T2 signal Higher apparent diffusion coefficient values than those of lymphoma
Cavernous hemangioma	Intraconal space, especially on the auricular side	High T2 signal Spot staining in the early phase, followed by a gradual spread of contrast enhancement in the dynamic contrast study
Pleomorphic adenoma, malignant salivary gland tumors	Lacrimal gland	Pleomorphic adenoma: bone remodeling, Well-defined margin Adenoid cystic carcinoma: bone destruction, Perineural spread

Notably, 72% of the primary orbital tumors are benign, and 28% are malignant [29]. Idiopathic orbital inflammation is the most common benign tumor, followed by Immunoglobulin G4-related disease (IgG4-RD), cavernous hemangiomas, and pleomorphic adenoma [29]. Lymphoma is the most common malignancy, followed by adenoid cystic carcinoma and solitary fibrous tumor [29]. In this article, we describe the clinical and imaging features of these common orbital tumors.

### IgG4 related disease

IgG4-RD is a systemic disease characterized by the abundant infiltration of IgG4-positive plasma cells and lymphocytes, leading to fibrosis and organ dysfunction. In the orbit, the lacrimal gland is the most common site of IgG4-RD, and it occurs at various sites, such as the external ocular muscles, eyelids, and orbital fat tissue. Serum IgG4 levels are elevated (> 135 mg/dL) and are included in the diagnostic criteria. Pathological and imaging findings are also included in the diagnostic criteria. Symptoms often include painless progressive proptosis, diplopia, and visual field disturbances. IgG4-RD is usually treated with steroid therapy.

**Fig. 8:**
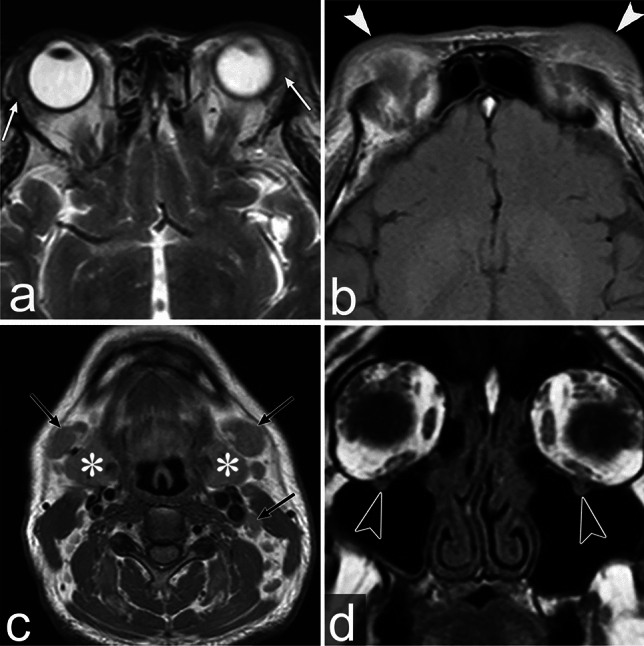
IgG4-related disease of a man in his 40s with left eyelid swelling for 1 year. **a**, **b** Axial T2 weighted images (WI) show bilateral lacrimal gland swelling (white arrows) and bilateral eyelid swelling (white arrowheads). **c** Axial T1WI shows bilateral cervical lymphadenopathy (black arrows) and bilateral submandibular gland swelling (asterisks). **d** Coronal T1WI shows bilateral infraorbital nerve enlargement (black arrowheads)

CT imaging typically shows swelling of the involved organ and homogeneous soft tissue density [30]. Owing to its increased cellularity and amount of fibrosis, it shows relatively low signal intensity on T2WI [30]. It shows a low signal intensity on T1WI with homogeneous enhancement [30]. Perineural spread may occur, especially involving the trigeminal nerve, particularly the infraorbital and frontal nerves, which is a characteristic finding of IgG4-RD (Fig. 8) [30].

Extraorbital head and neck lesions are observed in the salivary glands, lymph nodes, larynx, pituitary gland, thyroid gland, sinonasal cavities, and dura mater (Fig. 8) [30]. The presence of these lesions contributes to the diagnosis of IgG4-RD. When IgG4-RD is suspected, full-body examinations, such as CT, are needed to detect other organ lesions.

### Idiopathic orbital inflammation

Idiopathic orbital inflammation is a benign, noninfectious inflammatory disease of unknown cause. It is also known as inflammatory pseudotumor. Based on the location of the lesion, it is mainly classified into anterior, diffuse, apical or posterior, myositis, and dacryoadenitis types [31]. It is often unilateral and incidence of bilateral is 8–20% [31]. It predominantly occurs in patients in their 50s with no sex difference. It typically presents with orbital pain with acute to subacute onset. Depending on the type of lesion, various symptoms, such as eyelid swelling, conjunctival injection, proptosis, ocular motility restriction, and visual loss, may occur.

**Fig. 9:**
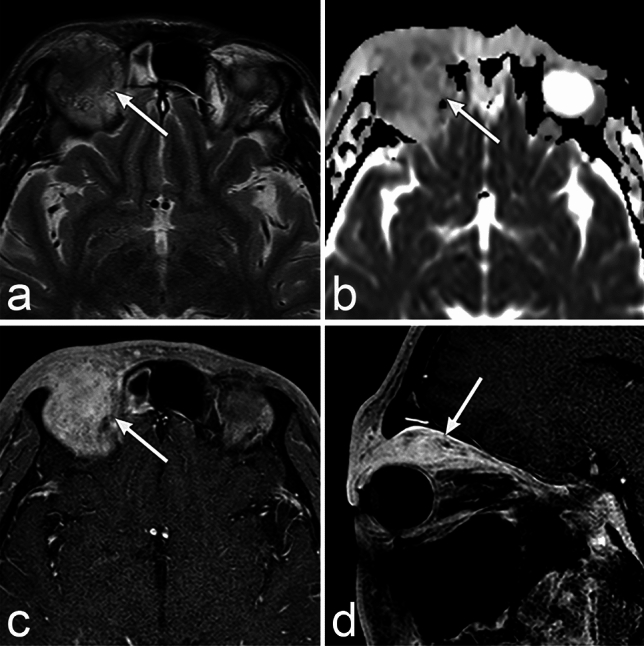
Idiopathic orbital inflammation of a man in his 40s with acute right orbital pain. **a** Axial T2 weighted image (WI) shows heterogeneous signal intensity mass above the right eyeball (arrow). **b** The apparent diffusion coefficient value of the mass (arrow) is 1.44 × 10^–3^ mm^2^/s. **c** Axial contrast-enhanced (CE) T1WI with fat suppression shows marked enhancement in the mass (arrow). **d** The mass infiltrates orbital fatty tissue, lacrimal gland, upper eyelid, superior rectus muscle, and superior oblique muscle of the right eye (arrow) on sagittal CE T1WI with fat suppression

Idiopathic orbital inflammation presents with mass formation in various orbit areas (Fig. 9). The mass shows density similar to that of the extraocular muscle on CT and low to iso signal intensity compared with the extraocular muscle on T1WI and T2WI. The mass shows variable contrast enhancement, marked enhancement in acute lesions [31]. The ADC value of idiopathic orbital inflammation is 1.49 × 10^–3^ mm^2^/s, which is higher than that of malignant lymphoma (0.70 × 10^–3^ mm^2^/s) and useful for differentiating between them [32]. In the apical type, it may extend from the superior orbital fissure to the middle cranial fossa and cavernous sinus. In the myositis type, the extraocular muscles are infiltrated. Involvement of the extraocular tendon and surrounding fatty tissue is a differentiating feature of thyroid orbitopathy [33].

### Cavernous hemangioma

Cavernous hemangioma, classified as a low-flow venous malformation rather than a true tumor, predominantly affects women, typically in their 40s [28]. It tends to grow very slowly; however, it can lead to its enlargement. Patients usually present with painless proptosis, with over half experiencing visual field defects due to optic nerve compression or altered blood flow caused by the lesion [28, 34]. Other symptoms include vision loss, diplopia, and pain. Cavernous hemangioma is also often identified incidentally during imaging [35]. Tumor resection is indicated in symptomatic cases [36].

**Fig. 10:**
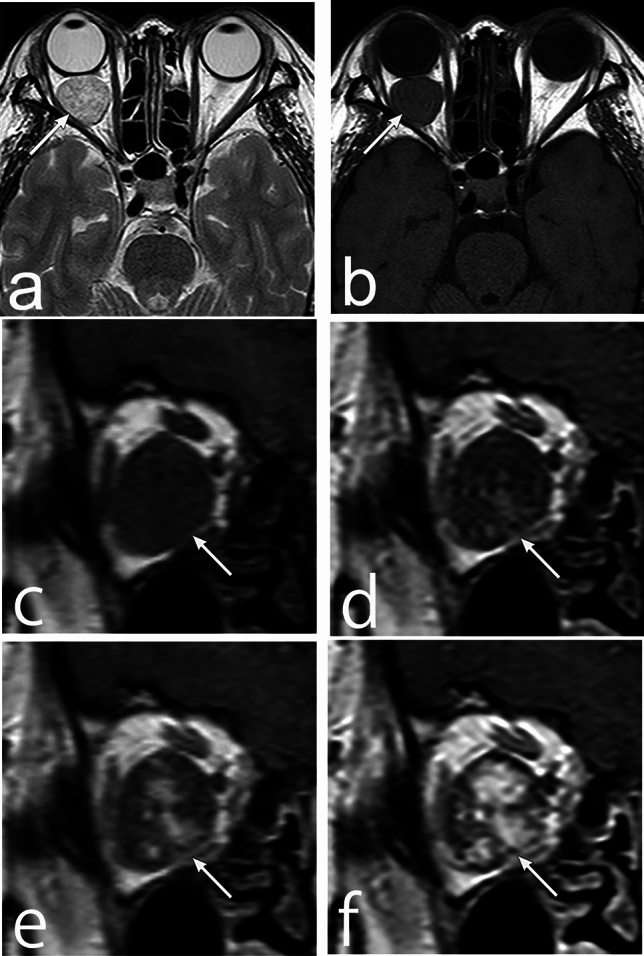
Cavernous hemangioma of a woman in her 50s with right proptosis. **a** Axial T2 weighted image (WI) shows high signal intensity and well-defined mass (arrow) in the left intraconal space. **b** The mass (arrow) shows low signal intensity on T1WI. **c**–**f** Dynamic contrast study (coronal T1WI with fat suppression) at precontrast, 37 s, 70 s, and 103 s, respectively, shows spot staining in the early phase, with the gradual spreading of the contrast enhancement in the mass (arrow)

Cavernous hemangiomas predominantly occur in the intraconal space, particularly on the auricular side, presenting as well-defined round or oval masses [35]. CT imaging typically shows a homogeneous-density mass with a few phleboliths [28]. As it grows, it compresses the surrounding tissues, such as the extraocular muscles, optic nerve, and bone, causing bone remodeling. On MRI, it appears iso-signal intensity with the muscle on T1WI [35], while T2WI shows more intense signal intensity than that of the muscle and may visualize internal septal structures (Fig. 10) [35]. Dynamic contrast studies exhibit spot staining in the early phase, followed by a gradual spread of contrast enhancement [37]. Schwannomas, lymphatic malformations, solitary fibrous tumors, varices, and meningiomas are among the differential diagnoses; however, dynamic contrast study findings also aid in distinguishing them.

### Pleomorphic adenoma of the lacrimal gland

Pleomorphic adenomas are the most common benign lacrimal gland tumors [38]. The lacrimal gland is divided into the palpebral and orbital parts by the tendon of the levator palpebrae superioris muscle. Pleomorphic adenomas predominantly occur in the orbital part. Patients usually present with painless unilateral proptosis [38]. Incomplete tumor resection or tumor rupture can lead to tumor recurrence or malignant transformation [28]. Therefore, when a clinical and imaging diagnosis of pleomorphic adenoma is made, a complete resection, including the tumor capsule, is performed without a biopsy [28].

**Fig. 11:**
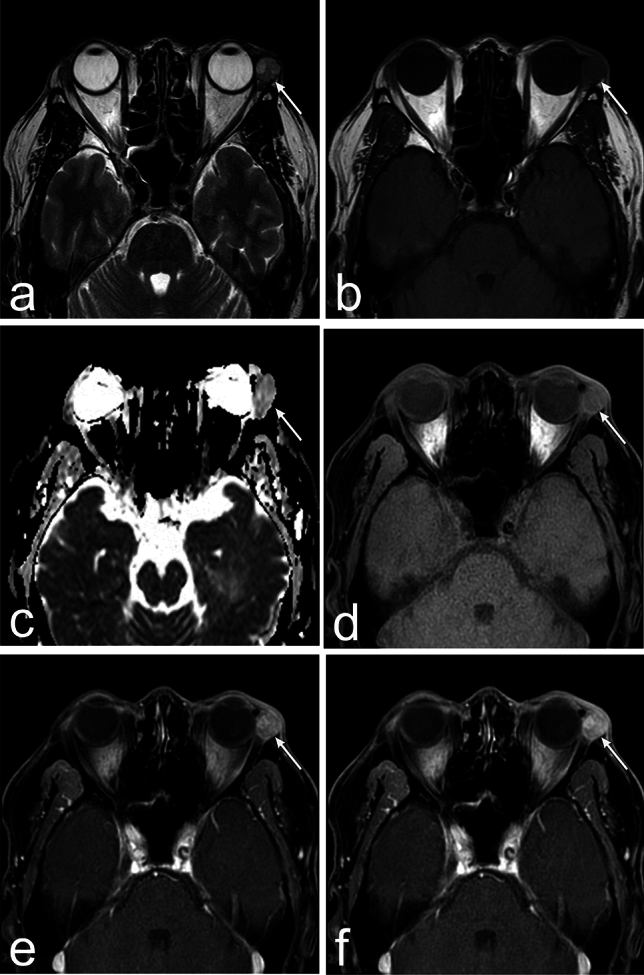
Pleomorphic adenoma of the lacrimal gland of a man in his 50s with left eyelid swelling. **a** Axial T2 weighted image (WI) shows heterogeneous high signal intensity and well-defined mass (arrow) in the left lacrimal gland. **b** The mass (arrow) shows low signal intensity on T1WI. **c** The apparent diffusion coefficient value is high in the mass (arrow). **d**–**f** Dynamic contrast study (axial T1WI with fat suppression) at precontrast, 40 s, and 160 s, respectively, shows gradual enhancement in the mass (arrow)

Pleomorphic adenomas present with a well-defined, round, or lobulated appearance, with tumor calcification being rare [38]. Due to slow growth, they may cause bone remodeling [38]. On MRI, they exhibit low signal intensity on T1WI and high signal intensity on T2WI (Fig. 11) [38]. However, with progression, they may show heterogeneity on CT and MRI owing to cystic degeneration, necrosis, and hemorrhage within the tumor [39]. Dynamic contrast studies show gradual enhancement.

### Adenoid cystic carcinoma of the lacrimal gland

Adenoid cystic carcinoma is the most common malignant epithelial tumor of the lacrimal gland [38]. It most commonly occurs in patients in their 40s and typically presents as a painful lacrimal gland mass [38]. It develops invasively and tends to develop perineural spread [28, 38]. Patients present with pain and sensory disturbances due to perineural spread [38]. Distant metastases are most prevalent in the lungs [38]. Treatment commonly involves surgical resection; however, complete resection is difficult and easily recurrent [40]. The combination of surgery with intra-arterial chemotherapy and adjuvant chemoradiotherapy improves the prognosis [40].

**Fig. 12:**
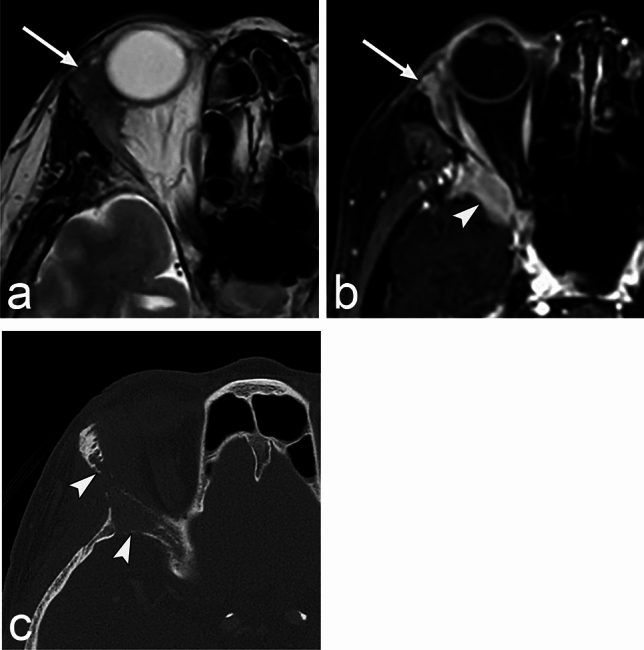
Adenoid cystic carcinoma of the lacrimal gland of a woman in her 70s with pain in the right eye. **a**, **b** MRI shows an ill-defined enhanced mass (arrow) in the right lacrimal gland. The mass shows iso-signal intensity compared to the brain cortex on the T2 weighted image. The mass invades the sphenoid bone (arrowhead). **c** CT shows bone destruction in the zygomatic and sphenoid bones (arrowheads)

In the early stages of the disease, it is difficult to distinguish it from pleomorphic adenoma on imaging. It is characteristic that adenoid cystic carcinoma shows bone destruction and perineural spread in the advanced stage of the disease (Fig. 12) [41]. Bone invasion occurs in approximately 80% of cases [42]. It shows diffuse enhancement and can be accompanied by calcification on CT. MRI shows low signal intensity on T1WI and iso-to-high signal intensity on T2WI compared with the brain cortex [38].

### Malignant lymphoma

Malignant lymphoma can occur in any part of the orbit, including the lacrimal glands, extraocular muscles, eyelids, and conjunctiva. Orbital primary lymphoma is often localized to the orbit. Mucosa-associated lymphoid tissue lymphoma is the most common histological type; diffuse large B-cell lymphoma, follicular lymphoma, and mantle cell lymphoma can also occur [43]. The most common symptoms are proptosis, a palpable mass, eye irritation, and ptosis [44].

**Fig. 13:**
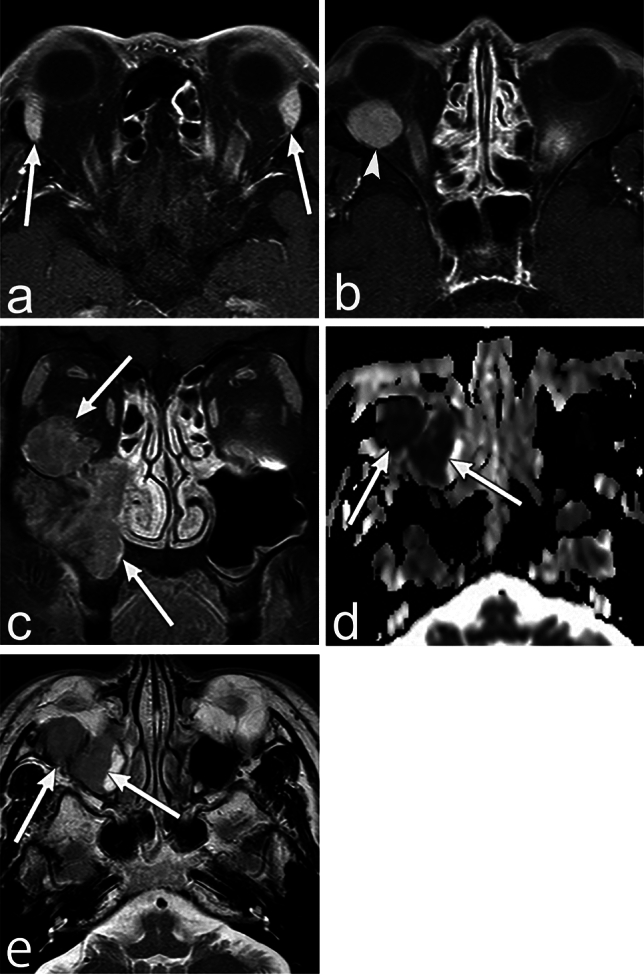
Diffuse large B-cell lymphoma of a man in his 60s with right eye proptosis since 1 week (**a**, **b**) Axial contrast-enhanced (CE) T1 weighted images (WI) with fat suppression show bilateral lacrimal gland swelling (arrows) and enhanced mass behind the right eyeball (arrowhead). **c** Coronal CE T1WI with fat suppression shows an enhanced mass in the right maxillary sinus infiltrating the right orbit (arrows). **d** The mass (arrows) shows a restricted diffusion apparent diffusion coefficient (ADC) value of 0.55 × 10^–3^ mm^2^/s) on the ADC map. **e** The mass (arrows) shows intermediate signal intensity on T2WI

Orbital lymphoma typically extends along preexisting structures without bone erosion on CT and MRI [28]. CT imaging reveals relatively high density and homogeneous enhancement [28], while MRI shows isointensity relative to the brain on T1WI, iso-to-high intensity relative to the brain on T2WI, and variable enhancement (Fig. 13) [28]. Lesions involving the lacrimal gland appear as swellings. Neoplastic lesions such as pleomorphic adenoma, adenoid cystic carcinoma, sarcoidosis, and IgG4-RD are differential diagnoses. Lymphoma exhibits a lower ADC value of 0.54 × 10–3 mm^2^/s compared with benign lymphoproliferative diseases, such as IgG4-RD, which have an ADC value of 0.81 × 10–3 mm^2^/s, aiding in differential diagnosis (Fig. 13) [46].

## Conclusion

Understanding the predilection sites and characteristic imaging findings of skull base and orbital tumors can help in diagnosis and guide subsequent management decisions.
